# Fibrodysplasia Ossificans Progressiva: Report of a Case and Review of Articles

**Published:** 2011-09-25

**Authors:** J. Hashemi, A. Shahfarhat, A. Beheshtian

**Affiliations:** 1 Associate Professor, Department of Radiology, Imam Reza hospital, Mashhad University of Medical Sciences, Mashhad, Iran; 2 Associate Professor, Department of Pediatrics, Imam Reza hospital, Mashhad University of Medical Sciences, Mashhad, Iran; 3 Radiologist, Imam Khomeini Hospital, Daregaz, Iran

**Keywords:** Fibrodysplasia Ossificans Progressiva, Hallux Valgus, Autosomal Dominant, Bone

## Abstract

Fibrodysplasia ossificans progressiva (FOP) is a rare autosomal dominant disorder, characterized by painful swelling of muscles and connective tissue in the early years of life, consequently leading to ossification at a mean age of 4-5 years. We report FOP in a 2-year-old boy with palpable masses in the frontal and lower cervical paraspinal and left periscapular muscles.

He was born with hallux valgus. Despite this hallmark, he was referred to the hospital with the primary diagnosis of hematoma, but further investigation indicated FOP. The patient was discharged from the hospital with non steroidal anti-inflammatory drugs (NSAID) and education of the parents. The importance of this case was that in spite of the early occurrence of the typical presentation of FOP for more than one year and the fact that the patient's mother was a physician who had consulted with many specialists, the diagnosis had been missed.

This indicates that the general physicians, radiologists and other specialists' awareness and knowledge of FOP is insufficient.

## Introduction

Fibrodysplasia ossificans progressiva (FOP) or myositis ossificans progressiva also known as Munchmeyer disease is a rare autosomal dominant condition that begins in childhood, but most patients have a new mutation. Genetic analysis revealed that the FOP gene located on chromosome 4 and mutation in this gene causes an over expression of a bone morphogenetic protein (BMP4). This is characterized by proliferation of connective tissue in voluntary muscles, fascias, tendons and ligaments resulting clinically as painful swelling of the muscles and connective tissue. This swelling subsides, then after approximately 6 months or more, ossification starts at some sites at the mean age of 4-5 years. Eventually, heterotrophic bone formation interferes with normal movement of the patient and most of them are confined to a wheel chair by the third decade. Mortality is related to restrictive lung disease (inability to expand the chest). Congenital malformations which are characteristically observed in the great toes at birth in almost all cases of FOP are the diagnostic hallmark.

## Case Presentation

A 2-year-old boy presented with left periscapular and upper back paraspinal tender masses. He was referred from pediatricians to our hospital (Imam Reza) and subsequently admitted. He was the third child of 37-year-old mother delivered by cesarean section with good Apgar score, normal birth weight (4100gr), head circumference (HC) of 35 cm and length of 53 cm. His parents were non consanguineous and the similar disease was not observed in the family. The mother was a general physician and the father was an engineer. The patient’s sister and brother were healthy. In the past medical history, atopic dermatitis and lacrimal duct obstruction were seen at infancy. Growth and development was normal in the past two years.

**Fig. 1:**
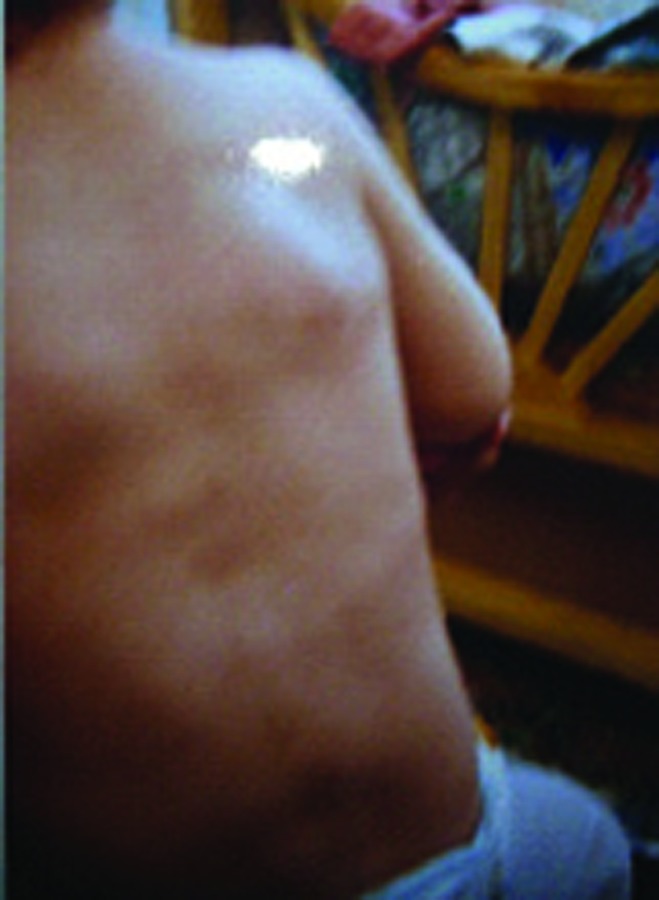
A one-year-old boy presenting with palpable masses in bilateral prescapular regions

Present disease began one year ago with painful swelling of the forehead after a mild trauma. Similar swelling in the arm and shoulders started later subsiding spontaneously. At this admission, he had paraspinal (upper thoracic and lower neck) and periscapular muscle swelling which was painful, tender and indurate, without redness and inflammation over the skin (Fig. 1). The shoulder girdle had normal ranges of motion.

**Fig. 2:**
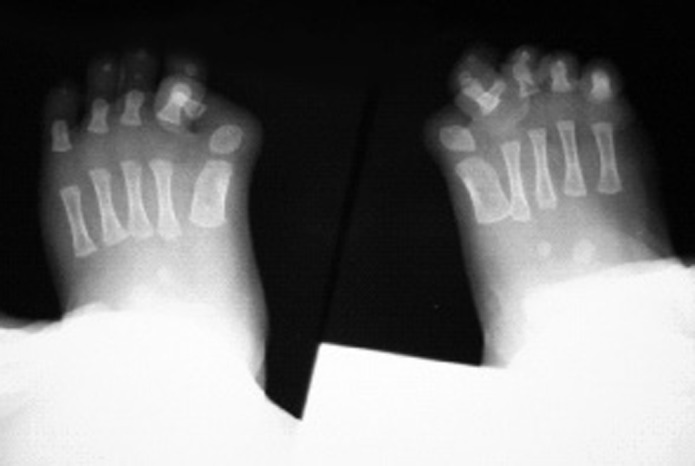
PA radiography of the feet shows bilateral hallux valgus congenital malformation as a hallmark of FOP

In physical examination of the limbs, bilateral hallux valgus was observed (Fig. 2).

He was referred with the primary diagnosis of multiple hematomas due to coagulation disorders, in which routine blood tests were normal twice and only hypochrome microcytic anemia was present. Ultrasonography showed solid hypoechoic masses with the largest diameter of 85 mm and thickness of 26 mm adhered to the scapula and spine. Sonographic differential diagnoses were mesenchymal tumor, bone tumor and aneurysmal bone cyst.

**Fig. 3:**
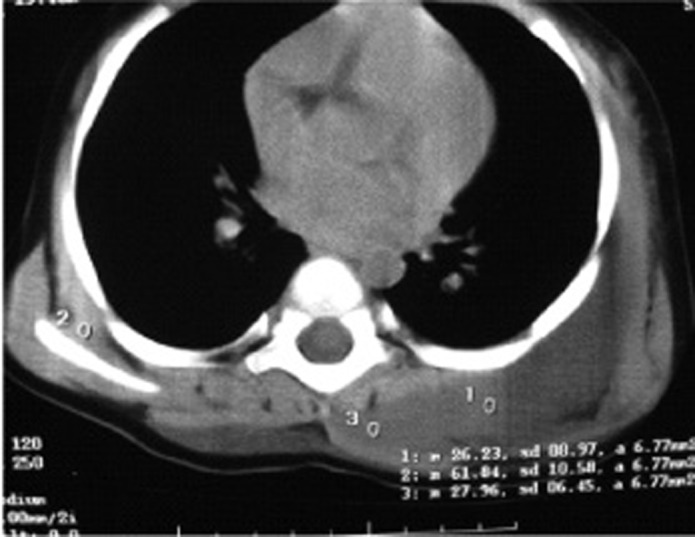
Thoracic CT-scan of this patient indicating soft tissue masses, 25 HU in density without bone formation and calcification in the prescapular and paravertebral regions. CT scan of the patient before calcification shows soft tissue masses in the posterior scapula region, prominent on the left side and a paraspinal soft tissue mass in the left upper thoracic region

The first CT scan indicated soft tissue masses, 25 Hounsfield Unit in density without bone destruction or calcification propounding mesenchymal or vascular masses (Fig. 3).

**Fig. 4:**
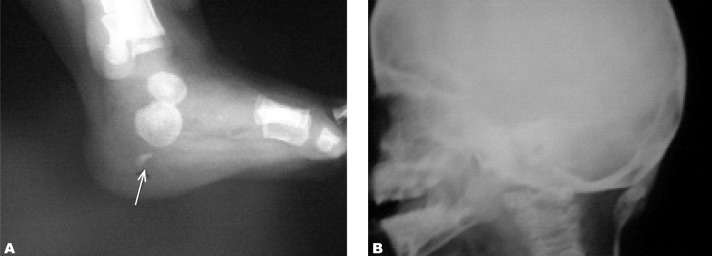
A. Foot and B. Neck radiography one year later at the age of two years show initiation of ossification in soft tissue masses

**Fig. 5:**
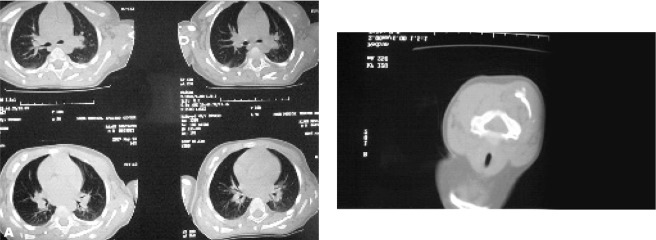
A. Thorax and B. Neck CT scan indicates soft tissue ossification in cervical and thoracic paravertebral soft tissue masses in the same case one year later at two years of age

**Fig. 6:**
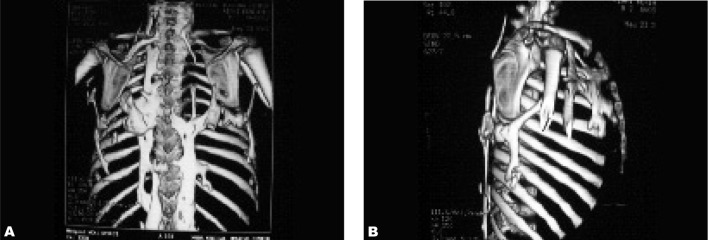
VR and 3D reconstructed CT of thoracic bones shows ossification in the paravertebral and periscapular region and the right arm in the same case at 2 years of age

In spite of typical clinical and imaging findings for the early stage of FOP such as the congenital malformation of bilateral hallux valgus as a hallmark in this step, the diagnosis was not made and an unnecessary biopsy was performed on the periscapular mass. It may have lead to catastrophic disability. The pathology report showed proliferation of fibro-connective tissue with large islets of compact cartilage cells and lacunars cells with blades and specula of bone and osteoblastic activation, compatible with fibrodysplasia ossificans progressiva. The patient was discharged with non steroid anti-inflammatory drugs. Almost one year later, extensive ossification was detected in foot and neck radiographies (Fig. 4), chest CT scan (Fig. 5) and reconstructed CT (Fig. 6).

## Discussion

### - History and Prevalence:

Gay patin first described this entity (FOP) in 1648 as a case who “turned to wood”. Prevalence is approximately 1/2.000.000 and over 700 cases have been reported in the world literature to date.[1][2] FOP is a rare and disabling genetic condition characterized by:

1. Congenital malformation of the great toes and

2. Initial soft tissue swelling which spontaneously subsides, but leads to progressive heterotrophic ossification in specific anatomic patterns.[3] The patients are confined to a wheel chair by the third decade. [4]

### - Pathologic Base of FOP:

**Fig. 7:**
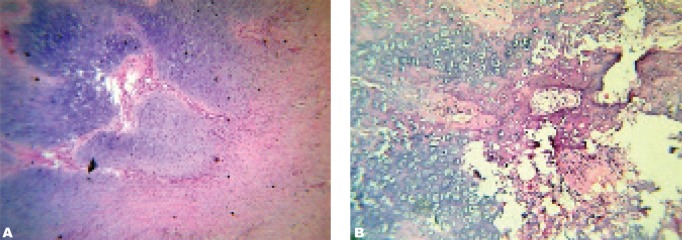
Pathologic feature shows fibro proliferative connective tissue with large islets of compact basophilic chondrocytes and lacunar cells surrounded by bone spicules and activated osteoblasts

 In our patient, pathologic assessment showed fibro proliferative connective tissue with large islets of compact basophilic chondrocytes and lacunar cells surrounded by bone specula and activated osteoblasts (Fig. 7).

### - Clinical and Radiological Findings:

Our case also started with this pattern, soft tissue swelling in the forehead with mild trauma. One year later, spontaneous swelling occurred in the shoulder, periscapular regions and the neck. The mean age of symptom onset was 2.5 years (some in the first year and most of them between 2 and 5).[5][6][7]

The radiological evidence of ectopic ossification is not usually detected until 6 months (sometimes up to one year), after the appearance of masses so the mean age at onset of ossification has been reported as 5 years. Bony bridge causes skeletal contracture (the lumps decrease in size after a few weeks, but the inflammatory tissue which is replaced first by cartilage and then by bone causes restricted joint movement) [1][3]

In our case, the onset of symptoms with the typical anatomic pattern occurred at one year of age and spread at the second year. Ossification in the radiologic exam and CT scan was detected. In addition, ossification of thoracic soft tissue causes scoliosis and restrictive pulmonary disease. As a result of this event, the patient will be predisposed to chest infection and consequently death in the third or fourth decade prevalently. [1][8]

### - Diagnosis:

FOP should be diagnosed as early as possible based on history, clinical and imaging findings. The guide of diagnosis is bilateral anomaly of the great toes present from birth, reported almost in 100% [5][6][9] of the patients. Therefore, clinical and radiological examination is important.

Roentgenogram aid demonstrates minor osseous dysmorphism such as hallux valgus, clinodactyly, microdactyly, phalangeal shortening, metacarpal and metatarsal shortening, shallow acetabulum, short widened femoral neck, medial cortex thickening of the tibia, long small vertebra, fusion and segmentation of the spine, increased incidence of exostosis and long bone metaphyseal widening.

When the ectopic ossification occurs, in plane radiography, bony bridges running from the bones to the muscles spreading also along the fascial planes are seen [3][9].

### - Ultrasonography:

 FUS of early lesions may show sonolucent soft tissue mass and then when ossification occurs, echogenic and shadowing mass is detected.[1][2]

### -CT Scan:

 In early disease before ossification, CT demonstrates fascia plane edema and swelling and mesenchymal mass like lesion in the muscle that moderately enhances with IV contrast. In late disease after ossification and calcification, these findings could be seen in the muscles, fascia and connective tissue, especially near the bones.[1][3]

### - MRI:

 Although the diagnosis of FOP is more difficult when ectopic ossification is not present, MRI finding of a preosseous lesion is sufficient for accurate diagnosis of FOP in early stage [5][9]. In T1 weighted images, only a mass effect with displacement of fascia plane and in T2 weighted images, a homogeneous soft tissue mass markedly enhanced with contrast is seen. [3]

Although other soft tissue tumors mimic this MRI finding, MRI appearance of spreaded masses along the fascial planes is the single feature that confirms the diagnosis. Late MRI shows low T1 and T2 signal intensity associated with fibrous or calcified tissue.

### Bone Scan:

Bone scintigraphy with TC-MDP demonstrates heterotrophic ossification in the early stage and helps in the assessment of the extent and progression of the disease. [6]

In our patient, plain radiography, US and CT scan were performed. US showed hypoechoic soft tissue masses near the left scapula and spine corresponding to mesenchymal tumor or bone tumor and CT scan showed soft tissue masses with 25 HU in density that adhered to the bone without bone distraction or obvious calcification, propounded mesenchymal or vascular masses

### - Differential Diagnosis:

In the early phase, these patients may be misdiagnosed with malignant tumors especially sarcoma, rhabdomyosarcoma, aggressive fibromatosis, post traumatic myositis, lymphadenopathy, TB, scleroderma, dermatomyositis of childhood, RA, nodular fasciitis and Klippel-Feil syndrome. [1][5][8][9][10]

In the late phase, the differential diagnoses are osteosarcoma, progressive osseous heteroplasia (POH), Albright hereditary osteodystrophy (AHO), osteoma cutis, ankylosing spondylitis, Still's disease, calcinosis interstitialis ossificans, Weber-Christian disease, pseudohypoparathyroidism, hypervitaminosis D, traumatic myositis ossificans and multiple exostosis. [1][8][10]

FOP has many differential diagnoses. This disease may be misdiagnosed in the early stage most importantly and most commonly as sarcoma and other cancers, aggressive fibromatosis and vascular masses.

In summary, in review of case series and case reports and articles of diagnostic errors, and our case, we conclude that FOP is often misdiagnosed and the correct diagnosis happens very late. Early diagnosis prevents catastrophic harmful diagnostic and treatment procedures. For early correct diagnosis we propose:

1. Family physicians, pediatricians and radiologists should be aware of the early feature of FOP before development of heterotrophic ossification. The most important and best key is great toe malformation which must be noticed clinically and radiologically as an early pattern of involvement.

2. Early clinical and radiological diagnosis must lead to an MRI study or molecular genetic study, which depends on the accessibility and cost. Both of them proved the diagnosis.

## References

[1] Subasree R, Panda S, Pal PK, Ravishankar S (2008). An unusual case of rapidly progressive contractures: case report and brief review. Ann Indian Acad Neurol.

[2] van der Meij EH, Becking AG, van der Waal I (2006). Fibrodysplasia ossificans progressiva. An unusual cause of restricted mandibular movement Oral Dis.

[3] Hagiwara H, Aida N, Machida J, Fujita K, Okuzumi S, Nishimura G (2003). Contrast-enhanced MRI of an early preosseous lesion of fibrodysplasia ossificans progressiva in a 21-month-old boy. AJR Am J Roentgenol.

[4] Gonçalves AL, Masruha MR, de Campos CC, Delai PL, Vilanova LC (2005). Fibrodysplasia ossificans progressiva: case report. Arq Neuropsiquiatr.

[5] Kaplan FS, Xu M, Glaser DL, Collins F, Connor M, Kitterman J (2008). Early diagnosis of Fibrodysplasia ossificans progressiva. Pediatric.

[6] Kitterman JA, Kantanie S, Rocke DM, Kaplan FS (2005). Iatrogenic harm caused by diagnostic errors in fibrodysplasia ossificans progressiva. Pediatrics.

[7] Smith R (1998). Fibrodysplasia (myositis) ossificans progressiva. Clin Orthop Relat Res.

[8] Vashisht R, Prosser D (2006). Anesthesia in a child with fibrodysplasia ossificans progressiva. Paediatr Anaesth.

[9] Lasry F, Touki A, Abkari A, Khalifa HH (2005). A rare cause of painful cervical swelling: myositis ossificans progressiva in childhoods. Report of a case. Joint Bone Spine.

[10] Mahajan S, Dang H, Gupta RK (2008). Myositis ossificans progressiva. Int J Orthop Surg [Serial online].

